# A Key to Every Imposture and Villainy

**Published:** 1869-12

**Authors:** 


A KEY TO EVERY IMPOSTURE AND VILLAINY.—"To surround everything, however monstrous and ridiculous, with an air of mystery, is to invest it with a secret charm, and power of attraction, which, to the crowd, is irresistible. False priests, false prophets, false doctors, false patriots, false prodigies of every kind, veiling their proceedings in mystery, have always addressed themselves at an immense advantage to the popular credulity, and have been, perhaps, more indebted to that resource in gaining and keeping for a time the upper-hand of truth and common sense, than to any other half dozen items in the whole catalogue of imposture. Curiosity has been from the creation of the world a master passion. To awaken it, to gratify it by slight degrees, and yet leave always something in suspense, is to establish the surest hold that can be had on the unthinking portion of mankind."

In all ages the thought above, so beautifully clothed, has been the foundation-stone of every quack and quackery that has disgraced the name of medicine. Let every honest physician exert himself to rend any remaining veil of any department of his profession. Have no mystery about anything, and publish to every one that wherever there is mystery, there is wrong, and that whenever a doctor is mysterious, he is to that extent a rogue. Publish a condensed code; let every family have one. All the mystery about consultation that now so annoys good meaning, intelligent people, would instantly disappear."—*Jour. of Med. and Surgery.*

